# Fair Learning Environment and Financial Well-Being as Resources for Students’ Academic Engagement: Basic Psychological Need Satisfaction as a Common Motivational Pathway

**DOI:** 10.3390/bs16101806

**Published:** 2026-10-02

**Authors:** Tuğba Hoşgörür, Yılmaz İlker Yorulmaz

**Affiliations:** Department of Educational Administration, Faculty of Education, Muğla Sıtkı Koçman University, Kötekli, Menteşe, 48000 Muğla, Türkiye; t.hosgorur@mu.edu.tr

**Keywords:** academic engagement, basic psychological need satisfaction, higher education, fair learning environment, subjective financial well-being

## Abstract

Academic engagement is associated with the resources available to students, yet it remains unclear through which psychological processes resources of different origins are related to academic engagement. Bringing together Basic Psychological Needs Theory and the Study Demands–Resources framework, this study examined whether autonomy, relatedness, and competence satisfaction mediate the associations of an institutional resource, a fair learning environment, and a resource rooted outside the academic environment, subjective financial well-being, with academic engagement. The measures were first evaluated using data from 278 university students. The structural model, specifying the three needs as parallel mediators, was then tested in a subsequent, non-overlapping sample of 741 students in Türkiye. Both resources positively predicted all three need satisfaction dimensions, whereas academic engagement was predicted only by autonomy (*β* = 0.43) and competence satisfaction (*β* = 0.36); relatedness satisfaction made no significant unique contribution. Neither resource was directly associated with academic engagement. The total indirect effects were *β* = 0.31 for a fair learning environment and *β* = 0.13 for subjective financial well-being. The model explained 47.4% of the variance in academic engagement. Resources of different origin were indirectly associated with academic engagement through the same two needs, autonomy and competence satisfaction.

## 1. Introduction

The extent to which university students participate in their academic work with energy, dedication and absorption stands out as an important factor for learning outcomes and for persistence in higher education. This motivational state, defined by Schaufeli et al. (2002) as academic engagement, is moderately associated with achievement in school-age samples (H. Lei et al., 2018) and also sits at the center of the models that explain why students continue in higher education (Tight, 2020; Tinto, 2017). Engagement, however, is not a fixed personal disposition. It is a dynamic process, shaped by the student’s continuous appraisal of the conditions they find themselves in. Experiences such as the attitudes of teaching staff, involvement in decision-making and a sense of belonging to the academic community make up the institutional side of these appraisals (Lizzio et al., 2007; Strayhorn, 2019). The material circumstances a student lives in form another critical context, one that affects their access to the social and academic resources needed in this process (Herr et al., 2024; Moore et al., 2021). Different as their origins are, both sets of conditions have the potential to shape how supportive and sustainable students perceive their academic experience to be.

In motivation research, these two sets of conditions correspond to different types of resource. The Study Demands–Resources (SD-R) framework, adapted to higher education, proposes that student engagement is supported by both contextual and personal resources (Bakker & Mostert, 2024; Lesener et al., 2020; Salmela-Aro et al., 2022). On this account, contextual resources are the social, relational and organizational conditions the institution provides, whereas personal resources are what students bring with them into the academic setting. Students, however, also draw on resources that lie entirely outside the study environment. Bakker and Mostert (2024) note that students encounter demands and resources in other life domains as well, and a recent review of SD-R measures confirms that research so far has treated demands and resources as located within the study environment alone (Stoffels & Pienaar, 2026). The present study takes a fair learning environment as the contextual resource and subjective financial well-being as a resource of this second, less examined kind.

The literature includes studies linking justice perceptions to students’ engagement and well-being (Berti et al., 2010; Chory et al., 2017; Di Battista et al., 2014; Główczewski & Burdziej, 2023; Navarro-Abal et al., 2018), and, in much the same way, financial circumstances to indicators of academic performance and well-being (Heckman et al., 2014; Russell et al., 2025; Ye et al., 2021). While these two literatures have developed in parallel, both leave unanswered the question of how a resource comes to support engagement. For an institutional or material condition to turn into a motivational state for the student, it must first come to have a counterpart in the student’s inner world. Answering this question therefore requires a theory that establishes this connection between environmental conditions and the individual.

Basic Psychological Needs Theory (BPNT), one of the sub-theories of Self-Determination Theory (SDT; Ryan & Deci, 2017), offers a comprehensive framework for such an explanation, proposing that the conditions an individual encounters in a social context reach individual functioning through the satisfaction or frustration of basic psychological needs. The theory specifies three such needs, namely autonomy, competence and relatedness (Chen et al., 2015; Vansteenkiste et al., 2020). Moreover, this process may be sensitive not only to the contextual conditions the institution provides but also to the conditions students bring with them into the academic context (Bakker & Mostert, 2024). When the SD-R account of the resources that support engagement is considered alongside the BPNT account of the psychological processes through which these effects come about, an integrated framework emerges that explains how resources are converted into engagement.

This study proposes and tests a model in which students’ perceptions of a fair learning environment (a contextual resource) and their subjective financial well-being (a personal resource) reach academic engagement through the satisfaction of basic psychological needs. Its contribution is twofold. First, it tests whether need satisfaction functions as a common motivational process for two resources that differ in origin, one individual and the other institutional. Bakker and Mostert (2024) claim that study resources are motivating because they satisfy basic psychological needs. None of their propositions, however, concerns need satisfaction, and the claim has not been tested for a resource lying entirely outside the study context. The second contribution is that it treats subjective financial well-being as one of the ways in which social class is experienced in academic life, and asks whether material inequality shapes not only students’ capacity to participate in that life but also their motivation toward it.

### 1.1. Basic Psychological Needs Theory and Academic Engagement

BPNT treats the three psychological needs as distinct sets of experience, and their emergence is neither contingent on the frustration of the other needs nor derived from them (Vansteenkiste et al., 2020). Autonomy is a person’s volitional direction and endorsement of their own actions, competence is feeling capable and effective in their interactions with the environment, and relatedness is forming meaningful bonds with others and feeling that one belongs to a group (Ryan & Deci, 2017). All three are necessary for optimal functioning, and satisfaction of one does not compensate for the frustration of another. This distinctness is also empirically supported. In a study spanning four cultures, Chen et al. (2015) showed that the satisfaction of each need contributed uniquely to well-being and the frustration of each contributed uniquely to ill-being. In follow-up analyses, the same study also showed that which need makes a unique contribution varies with the outcome under consideration. Autonomy and relatedness contributed uniquely to life satisfaction, whereas the contribution of competence was not significant (Chen et al., 2015).

What makes the theory useful for educational research is that the needs can be treated as a mediating process explaining the link between environmental conditions and psychosocial outcomes. Educators’ attitudes, classroom interactions and the ways institutional structures relate to students are precisely the conditions that support or thwart need satisfaction (Vansteenkiste et al., 2020). In a comprehensive meta-analysis, Howard et al. (2025) found need-supportive teacher behaviors to be positively associated with student performance, engagement and well-being, and thwarting behaviors to be associated in the opposite direction. The intervention literature points to similar conclusions while also revealing a divergence among the needs. A meta-analysis of SDT-based educational interventions found consistent support for autonomy and competence but no significant overall effect for relatedness (Wang et al., 2024b). The same divergence appears at the level of prediction. Bureau et al. (2022) reported that once the variance shared among the needs is accounted for, competence explains the most unique variance in forms of academic motivation and relatedness the least.

Among these outcomes, academic engagement stands in a particularly close relation to need satisfaction. Students whose needs are met may approach their work with more energy and resilience, find more meaning in what they do, and become absorbed in their work more readily. These correspond to the three components that Schaufeli et al. (2002) identified in the engagement construct as vigor, dedication and absorption. The link between academic engagement and need satisfaction is also supported by empirical studies conducted in a range of samples and contexts. Karimi and Sotoodeh (2020) reported that satisfaction of all three needs, autonomy, competence and relatedness, positively and significantly predicted academic engagement among university students, with the lowest coefficient obtained for relatedness. Wang et al. (2024a) similarly found that need satisfaction mediated the relationship between teacher autonomy support and classroom engagement, and He et al. (2025) found that all three needs predicted engagement in online learning, with relatedness making the weakest contribution. Satisfaction of the three needs is accordingly expected to predict academic engagement positively.

### 1.2. A Fair Learning Environment as a Contextual Resource

Students’ judgments about the fairness of their institution can be expected to shape the orientation they take toward academic life. A fair learning environment rests on students’ evaluation of the practices they meet in classroom and institutional interactions, judged against criteria of equity, consistency, transparency, openness and interpersonal respect (Lizzio et al., 2007). Because the object of these judgments is the learning environment, perceived fairness is treated as a contextual resource. The organizational justice literature divides these evaluations into four dimensions. Distributive justice concerns the fairness of the outcomes and resources obtained; procedural justice the consistency, impartiality, correctability and openness to participation of decision-making and implementation processes; interpersonal justice whether individuals are treated with respect and dignity; and informational justice whether explanations of processes and decisions are provided adequately, promptly and honestly (Colquitt, 2001). In educational settings, however, students’ own judgments do not reproduce this fourfold distinction. Lizzio et al. (2007) obtained a two-factor solution, respectful partnership, which carries most of the interpersonal content, and systemic fairness, which concerns the operation of information and problem-solving procedures. Comparable convergences have been reported in other educational samples (Berti et al., 2010; Di Battista et al., 2014). The present study adopts this two-factor structure derived from work in educational organizations rather than the four-dimensional organizational model.

That respectful partnership carries the greater weight of the two suggests that what students register most keenly is how they are treated within the workings of the institution. This connects the fair learning environment to their sense of belonging. Indeed, Strayhorn (2019) defines belonging in college as the perception that one is supported, connected and, above all, valued by the campus community, and treats it as a basic need whose satisfaction depends on the surrounding environmental conditions. Tinto (2017) arrives at the same point from the persistence literature, arguing that belonging is shaped less by the number of interactions students have than by what those interactions communicate about whether they are valued. An environment in which academic staff regard students as respected partners, apply procedures consistently and keep problem-solving channels open conveys precisely this message. Seen this way, belonging appears not as an outcome standing alongside need satisfaction but as a relational experience closely aligned with relatedness satisfaction (Bohndick et al., 2025).

Empirical work supports this expectation while also giving indications that the route from fairness to engagement is less direct than it first appears. At the level of simple association, the patterns are clear. Perceptions of organizational justice correlate positively with academic engagement and negatively with academic burnout (Navarro-Abal et al., 2018). Studies that trace the connection more closely, however, show that the relationship does not operate directly. Di Battista et al. (2014) found that students’ overall sense of justice predicted both identification with the institution and compliance with its rules but was not directly related to voluntary participation, which emerged only among students who identified strongly with the institution. Główczewski and Burdziej (2023) showed that procedural and distributive fairness predicted academic engagement when entered on their own, but that both coefficients became non-significant once academic identification and perceived institutional legitimacy were introduced as serial mediators. These findings suggest that fairness may operate through intervening psychological processes in its association with engagement, providing a close precedent for the model tested here.

The path by which a fair learning environment reaches engagement can be explained by its functioning as a contextual resource that feeds need satisfaction. The respectful partnership dimension of Lizzio et al. (2007) may support the needs for autonomy and relatedness by granting students a voice in decision-making processes and positioning them as valued members of the academic community. The systemic fairness dimension may support the need for competence by allowing students to know where they stand through predictable, transparent and correctable procedures. Fairness is in this respect also a medium through which the institution conveys the message that it takes a student’s autonomy, capability and membership seriously. This expectation is not limited to the fairness literature. In a systematic review of the relationship between perceived teacher support and engagement in higher education, Prananto et al. (2025) identified mediation through basic psychological needs as one of the three principal explanatory patterns in this body of work. The present study extends this perspective by considering whether similar processes may also apply to conditions situated at the institutional level, alongside those operating at the interpersonal level. Studies testing the link between a fair learning environment and need satisfaction directly remain scarce, but the indirect evidence is consistent (Liu et al., 2024; Xin, 2022). On this basis, a fair learning environment is expected to predict the satisfaction of all three basic psychological needs positively.

### 1.3. Subjective Financial Well-Being as a Personal Resource

Students participate in academic life under different material circumstances. Financial circumstances are an important dimension of social class in higher education, shaping both the resources available to students and how they perceive their position relative to their peers. What appears to shape psychological functioning is how students evaluate their own socioeconomic position, with these subjective evaluations potentially carrying greater weight than objective income. Di Domenico and Fournier (2014), in a community sample of adults, found that subjective socioeconomic status predicted need satisfaction markedly more strongly than household income did (*β* = 0.33 against *β* = 0.12), and that income exerted much of its influence through the way it shapes a person’s subjective standing. Regional income inequality also predicted lower need satisfaction in its own right. This finding suggests that what unsettles psychological functioning is, alongside the material level itself, the relative deprivation that renders differences between people visible. Subjective financial well-being is accordingly treated in this study as one way in which social class is subjectively registered in academic life.

This framing also calls for a distinction concerning the nature of the resource. The SD-R literature counts financial problems among study demands (Bakker & Mostert, 2024). What is being measured here, however, is not the presence of hardship. The object of study is whether students consider their own financial situation adequate. Brüggen et al. (2017) draw an explicit distinction between financial well-being and financial stress and note that the two are not ends of a single continuum. On this distinction, low financial well-being points less to the presence of a demand than to the insufficiency of a resource. Subjective financial well-being is not, moreover, a single overall evaluation. Sorgente and Lanz (2019) isolate general subjective financial well-being in emerging adults as a cognitive component carrying the person’s overall evaluation of and feelings about their financial condition, and on the strength of its psychometric properties they recommend that it may be used on its own in studies concerned with the general aspect of the construct. It is in this form that it enters the present study.

One of the criteria by which a need qualifies as basic in Basic Psychological Needs Theory is that it can explain how supportive or damaging changes in a person’s environment translate into well-being (Vansteenkiste et al., 2020). Students’ material circumstances can be treated as one such environmental condition. Financial security may support autonomy by affording students the time and latitude to pursue what genuinely interests them, competence by providing the conditions under which skills can be developed, and relatedness by easing financially driven strain. Financial difficulty works in the opposite direction. In their study, Mani et al. (2013) showed that preoccupation with monetary concerns consumes attentional and executive resources. The way they define poverty is instructive as well. The definition rests not on an income threshold but on the gap between a person’s needs and the resources available to meet them, and therefore, in keeping with the approach adopted here, on a subjective appraisal. For a student carrying that load, the attention that can be directed toward the support the academic environment offers may diminish, and drawing on the support available may become more difficult.

The influence of material circumstances on academic motivational processes has also been observed in university student samples. Ye et al. (2021) found that life satisfaction mediated the association between social support and academic burnout among university students, and that the strength of this indirect association varied according to students’ socioeconomic status. Heckman et al. (2014) reported that one of the two most salient sources of stress among university students was lacking the financial means to participate in the same activities as their peers, while students with higher financial self-efficacy and greater optimism about the future reported significantly lower levels of financial stress.

The influence of material circumstances can extend beyond the cognitive resources a student is able to devote to academic life and the practical means available to them. Among low-income, first-generation youth in the United States, a sense of belonging at school predicted whether they went on to enter higher education, whereas supportive relationships with adults did not (Lecy, 2021). If the material and the relational are already entangled at the point of access, this entanglement can be expected to persist after entry. Financial insecurity may therefore affect need satisfaction both through the pressure it places on attentional resources and through the way students evaluate the terms on which they participate in the academic community. The closest structural precedent for the pathway proposed here is the model of Di Domenico and Fournier (2014). In that model, need satisfaction mediated the relationship of subjective socioeconomic status, household income and regional income inequality with health complaints. Although the outcomes examined there concern physical health and the sample was drawn from the general adult population, the findings were considered important for the design of the present study in that need satisfaction occupies a mediating position between material circumstances and individual outcomes. On this basis, students’ subjective financial well-being is expected to predict the satisfaction of all three basic psychological needs positively.

### 1.4. Need Satisfaction as the Common Pathway

The framework that links contextual and personal resources to academic engagement emerged outside educational organizations. The Job Demands–Resources model separates the job demands that feed strain from the job resources that feed motivation (Bakker & Demerouti, 2017). Lesener et al. (2020) applied this distinction to higher education under the framework of Study Demands–Resources. In a sample of university students, they found that study resources strongly predicted student engagement. The framework was subsequently extended to include personal resources as well (Salmela-Aro et al., 2022). SD-R states that resources support engagement through a motivational process, but it does not clarify the content of that process or whether resources of different origin pass through the same one.

BPNT provides a more specific account of this motivational process. On this theory, resources feed optimal functioning and engagement to the extent that they support the satisfaction of the needs for autonomy, competence and relatedness. Whether a need counts as basic likewise depends on its capacity to explain how changes in the environment reach individual functioning, regardless of where those changes originate (Ryan & Deci, 2017; Vansteenkiste et al., 2020). Bakker and Mostert (2024) made this link explicit in integrating the two frameworks, proposing that the motivational effect of study resources operates through basic psychological need satisfaction. That the process is independent of origin also has empirical support. Rodríguez-Meirinhos et al. (2020) reported that the need satisfaction model showed measurement and structural invariance across low, middle and high socioeconomic status groups, and that the patterns of relations running from need satisfaction to well-being operated equivalently across these groups. In the model of Di Domenico and Fournier (2014), two antecedents of different kinds, one a perception and the other an objective material fact, subjective socioeconomic status and household income, reached the outcome through one and the same mediator.

What follows from this is the expectation that two resources of different origin may relate to academic engagement through the same psychological needs. The term common pathway here refers to the shared role of need satisfaction in linking both resources to engagement. Examining the three needs separately matters if this expectation is to be tested meaningfully. Where the three needs are combined into a single composite satisfaction score, it is not possible to determine whether the same need dimensions carry the associations of both resources with engagement. Treating the needs separately is not a choice arising solely from the design of the present study. The appropriateness of representing the needs by a single composite score has also been debated at the meta-analytic level (Van den Broeck et al., 2016). Chen et al. (2015) examined the unique contribution of each need by treating the three separately rather than as a composite need satisfaction indicator. Similarly, Rodríguez-Meirinhos et al. (2020) found that a structure representing the three needs as separate first-order factors fitted better than an alternative in which the needs were combined into a single composite satisfaction factor. In line with these theoretical and empirical grounds, autonomy, relatedness and competence are modeled in the present study as three separate and parallel mediators.

The two resources are likewise positioned as parallel antecedents in relation to each other. Although SD-R permits a specification in which personal resources mediate the effects of contextual ones (Salmela-Aro et al., 2022), it is not plausible to think of a fair learning environment and a student’s financial position as standing in a cause-and-effect relationship. The hypotheses are stated in a positive direction for all three needs, a choice resting on BPNT’s assumption that basic psychological needs are universal in character. The evidence reviewed above, however, does not provide a sufficiently clear basis for specifying differential hypotheses regarding the relative strength of the three need–engagement relationships. The general aim of this study is to examine whether autonomy, relatedness, and competence satisfaction account for the associations of a fair learning environment and subjective financial well-being with academic engagement, and whether the two resources are linked to engagement through the same need dimensions. The hypotheses tested are as follows:

**H1.**  *Perceived fair learning environment positively predicts autonomy, relatedness, and competence satisfaction.*

**H2.**  *Subjective financial well-being positively predicts autonomy, relatedness, and competence satisfaction.*

**H3.**  *Autonomy, relatedness, and competence satisfaction positively predict academic engagement.*

**H4.**  *Autonomy, relatedness, and competence satisfaction mediate the relationship between perceived fair learning environment and academic engagement.*

**H5.**  *Autonomy, relatedness, and competence satisfaction mediate the relationship between subjective financial well-being and academic engagement.*

**Figure 1:**
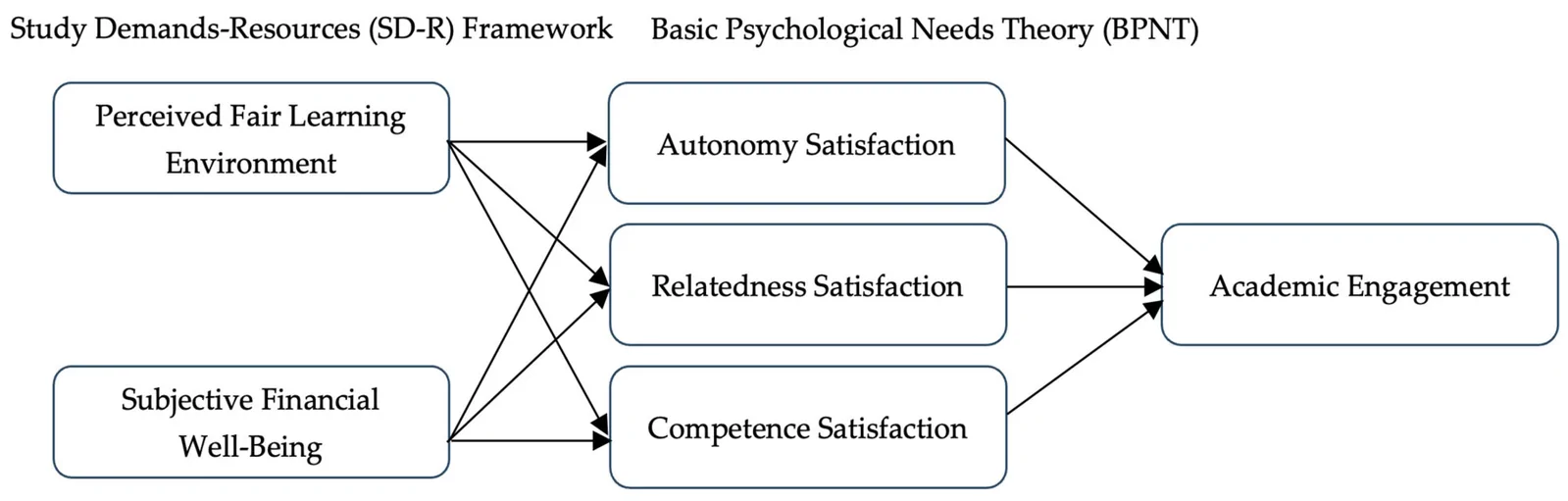
Conceptual Model Integrating the SD-R Framework and BPNT.

The conceptual model derived from these hypotheses is presented in Figure 1.

## 2. Materials and Methods

### 2.1. Research Design

This research employed a cross-sectional survey design and used two sequential, non-overlapping samples. The first sample was used to examine the psychometric properties of the measurement instruments among Turkish university students, whereas the subsequent sample was used to test the proposed structural model in which perceived fair learning environment and subjective financial well-being were specified as predictors of academic engagement through autonomy, relatedness, and competence satisfaction. The theoretical framework of the study integrated the SD-R framework, the higher-education adaptation of the JD-R model, with BPNT.

### 2.2. Participants and Procedure

The study was conducted in accordance with the Declaration of Helsinki and approved by the Educational Sciences Research Ethics Committee of Muğla Sıtkı Koçman University (protocol code. 260107; decision no. 126; approval date. 20 May 2026). Data were collected sequentially from two non-overlapping samples through online surveys using the same recruitment procedures. Prior to participation, digital informed consent was obtained from all participants by requiring them to explicitly select an agreement option. Only participants who provided informed consent were permitted to proceed with the questionnaire. To reach potential participants, the researchers used their institutional networks and collaborated with course instructors from various universities across Türkiye. After being informed about the purpose and procedures of the study, course instructors voluntarily assisted in disseminating the survey link to their students. The first sample comprised 278 university students and was used for the psychometric evaluation of the measurement instruments. After the psychometric adequacy of the measures had been confirmed, data collection continued, resulting in a subsequent sample of 755 students for structural model testing. Students in both samples were recruited from diverse higher education institutions and geographic regions across Türkiye.

In the first sample, each measure was evaluated separately; therefore, the final analytic sample size varied slightly across measure-specific analyses following outlier screening. Specifically, seven multivariate outliers were removed from the fair learning environment dataset, no outliers were detected in the financial well-being dataset, nineteen outliers (nine univariate and ten multivariate) were removed from the need satisfaction dataset, and ten outliers (seven univariate and three multivariate) were removed from the academic engagement dataset. Accordingly, the final analytic samples consisted of 271 participants for fair learning environment, 278 for subjective financial well-being, 259 for basic psychological need satisfaction, and 268 for academic engagement.

In the second sample, 14 cases (13 univariate outliers and one multivariate outlier) were removed from the dataset following data screening procedures. Accordingly, the final analytic sample consisted of 741 participants. The demographic characteristics of the two samples are presented in Table 1.

### 2.3. Measures

#### 2.3.1. Fair Learning Environment Scale

Perceived fair learning environment was assessed using the Turkish version of the Fair Learning Environment Scale. The original scale was developed by Lizzio et al. (2007), and its Turkish adaptation was conducted by Özer and Demirtaş (2010). Although the survey initially included the 16 original items, item FA9 was excluded from the analyses in line with the Turkish adaptation. The Turkish form used in the analyses consisted of 15 items across two dimensions: respectful partnership and systemic fairness. A sample item is “In this faculty, rules and procedures are applied consistently and fairly to all students.” Responses are rated on a five-point Likert-type scale ranging from 1 (disagree) to 5 (strongly agree), with higher scores indicating stronger perceptions of fairness in the learning environment.

In the original development study, principal axis factoring produced the two-factor solution described above, accounting for 51.6% of the total variance, with Cronbach’s alpha coefficients of 0.89 for respectful partnership and 0.77 for systemic fairness (Lizzio et al., 2007). In the Turkish adaptation, principal components analysis reproduced the same two-factor structure once item FA9 had been removed, the two factors together accounting for 44.71% of the variance, and Cronbach’s alpha coefficients were 0.81 for respectful partnership, 0.76 for systemic fairness and 0.87 for the total scale (Özer & Demirtaş, 2010). Neither study tested this structure with confirmatory factor analysis, and the factorial validity of the Turkish form was therefore re-examined in the first sample of the present study.

#### 2.3.2. Subjective Financial Well-Being Scale

Subjective financial well-being was assessed using the 10-item General Subjective Financial Well-Being dimension of the Turkish version of the Multidimensional Subjective Financial Well-Being Scale. The original scale was developed by Sorgente and Lanz (2019) to assess emerging adults’ subjective financial well-being, and cross-national evidence including Türkiye was provided by Sorgente et al. (2024). In the present study, this dimension was used to assess students’ overall subjective evaluation of their financial condition. A sample item is “I am satisfied with my current financial situation.” Responses are rated on a five-point scale ranging from 1 (completely false) to 5 (completely true), with higher scores indicating greater subjective financial well-being.

In the development studies, the five-factor structure of the scale was supported by confirmatory factor analysis (*χ*^2^_(265)_ = 1043.127, RMSEA = 0.075, CFI = 0.902, SRMR = 0.063), and the composite reliability of the general dimension was ω = 0.94 (Sorgente & Lanz, 2019). Evidence for the Turkish form comes from a nine-country study in which the five-factor model fitted the Turkish data well (*n* = 539; χ^2^_(265)_ = 800.18, RMSEA = 0.06, CFI = 0.92, SRMR = 0.05), the composite reliability of the general dimension was *ω* = 0.95, and approximate measurement invariance held across the eight countries in which the model was supported, allowing scores to be compared across them (Sorgente et al., 2024).

#### 2.3.3. Basic Psychological Need Satisfaction and Frustration Scale (BPNSFS)

Basic psychological need satisfaction was assessed using the satisfaction items of the BPNSFS. The scale was originally developed and validated by Chen et al. (2015) to assess both need satisfaction and need frustration. In the present study, the 12 satisfaction items from the English adult version of the BPNSFS manual were adapted to the academic context. During the adaptation process, item wording was revised to reflect university students’ academic experiences, including their courses, academic tasks, and university learning environment. The adapted form was reviewed by a panel of six experts in terms of linguistic clarity, content appropriateness, and suitability for the academic context. The adapted Turkish items and their English translations are provided in the Supplementary Information. The English translations were prepared through forward translation, back-translation, and expert committee review procedures to ensure conceptual equivalence. The English translations are provided for reference only and have not themselves been psychometrically validated. All analyses were conducted on the Turkish items. The scale assesses three dimensions of psychological need satisfaction: autonomy, competence, and relatedness. A sample item is “In my university education, I feel a sense of choice and freedom in the work I undertake.” Responses are rated on a five-point Likert-type scale ranging from 1 (strongly disagree) to 5 (strongly agree), with higher scores indicating greater psychological need satisfaction in the academic context.

In the original validation, a six-factor model distinguishing need satisfaction and need frustration for each of the three needs fitted the data well (Satorra–Bentler scaled *χ*^2^_(231)_ = 372.71, RMSEA = 0.03, CFI = 0.97, SRMR = 0.04), was cross-validated in an independent subsample, and showed metric invariance across the four countries studied. The internal consistency coefficients of the three satisfaction subscales ranged from 0.65 to 0.88 across countries (Chen et al., 2015). Because the items were adapted to the academic context in the present study, the factorial validity of the adapted form was examined in the first sample.

#### 2.3.4. Utrecht Work Engagement Scale–Student Form (UWES-SF)

Academic engagement was assessed using the Turkish nine-item short form of the Utrecht Work Engagement Scale-Student Form (UWES-SF) adapted by Çapri et al. (2017). The scale assesses students’ engagement with academic work across three dimensions: vigor, dedication, and absorption. A sample item is “I get carried away when I study.” Responses are rated on a five-point Likert scale ranging from 1 (never) to 5 (always), with higher scores indicating greater academic engagement.

In the Turkish adaptation, confirmatory factor analysis supported the three-factor structure of the nine-item form (*χ*^2^/df = 3.62, RMSEA = 0.066, CFI = 0.99, NNFI = 0.98, SRMR = 0.035), item-total correlations ranged from 0.32 to 0.62, and Cronbach’s alpha coefficients ranged from 0.72 to 0.88 across the subscales and the total score (Çapri et al., 2017). That adaptation study was conducted with high school students, and the factorial validity of the form in a university sample was therefore examined in the first sample of the present study.

### 2.4. Data Analysis

Prior to the main analyses, reverse-scored items in the Fair Learning Environment scale (Items 8, 12, and 15) and the Subjective Financial Well-Being scale (Items 2 and 5) were recoded before scale scores were computed. The data were then screened for missing values, normality, and univariate and multivariate outliers. Univariate outliers were identified using standardized *z* scores, with observations exceeding ±3.00 considered as potential outliers (Hair et al., 2019). Multivariate outliers were assessed using Mahalanobis distance across the variables included in each screening analysis, with a conservative significance threshold of *p* < 0.001 used to identify potential multivariate outliers (Hair et al., 2019). In the first sample, because the measures were evaluated independently, univariate outlier screening was conducted separately for each measurement instrument at the dimension level. In the second sample, univariate screening was conducted at the scale-score level. In both samples, multivariate outliers were assessed using all variables included in the respective analysis. As a sensitivity check, the structural model was also re-estimated using the full second sample before outlier exclusion to assess the robustness of the principal structural findings.

The normality of the data was assessed through skewness and kurtosis values. Following Schumacker and Lomax (2010), skewness and kurtosis values between −1.5 and +1.5 were considered indicative of acceptable normality. Descriptive statistics and Pearson correlation coefficients were calculated to examine the relationships among the study variables. Following Schober et al. (2018), correlation coefficients were interpreted as negligible when below 0.10, weak between 0.10 and 0.39, moderate between 0.40 and 0.69, strong between 0.70 and 0.89, and very strong at 0.90 or above. Multicollinearity among the study variables was examined using tolerance index and variance inflation factor values. Following Kline (2016), variance inflation factor values above 10 and tolerance index values below 0.10 were considered indicative of multicollinearity. Because all measures were self-reported and collected in a single session, Harman’s (1976) single-factor test was conducted as a preliminary indication of common method bias. The test is recognized as a limited diagnostic and was interpreted accordingly.

All analyses were conducted using IBM SPSS Statistics 22 and AMOS 22. In the first sample, a separate CFA was performed for each measurement instrument using maximum likelihood estimation to examine the factorial validity of the measures. Internal consistency was assessed using Cronbach’s alpha (*α*) and McDonald’s omega (*ω*) coefficients. In the second sample, the measurement model was first evaluated using CFA. The measurement model included 27 indicators: fair learning environment and academic engagement were represented by their respective dimension scores, whereas subjective financial well-being and the three need satisfaction constructs were represented by their individual items. Modification indices were inspected as part of the measurement-model evaluation, and robustness checks were conducted to assess whether any model specifications materially affected the substantive structural results. Subsequently, structural equation modeling (SEM) was employed to test the hypothesized relationships among perceived fair learning environment; subjective financial well-being; autonomy, relatedness, and competence satisfaction; and academic engagement. The direct paths from fair learning environment and financial well-being to academic engagement were also freely estimated to assess residual direct associations. Because autonomy, relatedness, and competence satisfaction represent conceptually related dimensions of basic psychological need satisfaction, the disturbances of these three endogenous constructs were allowed to covary in the structural model.

Model fit was evaluated using the following criteria. A *χ*^2^/*df* ratio 5 or lower was considered indicative of an acceptable fit (Wheaton et al., 1977). SRMR values of 0.08 or lower were considered indicative of acceptable fit (Hu & Bentler, 1999; Schreiber et al., 2006). RMSEA values below 0.05 were interpreted as indicating good fit, whereas values between 0.05 and 0.08 indicated acceptable fit (Schermelleh-Engel et al., 2003). For CFI and TLI, values above 0.90 were considered indicative of acceptable fit, whereas values of 0.95 or higher indicated good fit (P. W. Lei & Wu, 2007; Schermelleh-Engel et al., 2003; Schreiber et al., 2006).

The mediating roles of autonomy, relatedness, and competence satisfaction were examined using bias-corrected bootstrap confidence intervals based on 5000 resamples. Indirect effects were considered statistically significant when the 95% confidence interval did not include zero.

## 3. Results

The results are presented in two parts. First, the psychometric evaluation of the measurement instruments is reported. Second, the measurement and structural model results are presented.

### 3.1. Psychometric Evaluation of the Measurement Instruments

In the first sample, the psychometric properties of the study measures were evaluated through descriptive statistics, normality assessments, confirmatory factor analyses, and reliability analyses. Table 2 presents the descriptive statistics, skewness and kurtosis values, fit indices, and reliability coefficients for each measure.

As shown in Table 2, skewness and kurtosis values for all measures were within the acceptable range, supporting univariate normality. The CFA results indicated acceptable to good model fit across the four measures. In addition, Cronbach’s alpha and McDonald’s omega coefficients ranged from 0.874 to 0.957, indicating satisfactory internal consistency reliability. These findings supported the use of the measurement instruments in the subsequent structural model analyses.

### 3.2. Measurement and Structural Model Results

The results are presented in three sections: preliminary and descriptive analyses, measurement model evaluation, and structural model testing.

#### 3.2.1. Preliminary and Descriptive Analyses

The final analytic sample consisted of 741 participants. All study variables met the prespecified normality criterion, and the tolerance index and variance inflation factor values indicated no multicollinearity concerns (Table 3). Regarding common method bias, Harman’s single-factor test showed that the highest variance explained by a single factor was 26.61%, which was below the commonly used 50% threshold. However, because the single-factor test is a limited diagnostic, this finding was treated only as a preliminary indication and does not rule out the possibility of common method variance.

As shown in Table 3, all study variables demonstrated satisfactory internal consistency, with Cronbach’s alpha and McDonald’s omega coefficients ranging from 0.85 to 0.96. Pearson correlation analyses revealed that all study variables were positively and significantly interrelated. The observed correlations ranged from weak to moderate in magnitude (*r* = 0.15 to 0.56). Overall, these preliminary checks indicated that the dataset was suitable for confirmatory factor analysis and structural equation modeling.

#### 3.2.2. Evaluation of the Measurement Model

Prior to testing the hypothesized structural relationships, a CFA was conducted to evaluate the measurement model, in which autonomy, relatedness, and competence satisfaction were specified as distinct but correlated latent constructs. The initial measurement model, without correlated residuals, demonstrated acceptable fit to the data, *χ*^2^_(309)_ = 1136.186, *χ*^2^/df = 3.67, RMSEA = 0.060, SRMR = 0.042, CFI = 0.945, and TLI = 0.937. Inspection of the modification indices indicated a residual association between items 3 and 4 of the subjective financial well-being measure. Because both items refer to students’ satisfaction with their current financial situation, allowing their residuals to covary was considered substantively justifiable and the covariance was therefore added to the measurement model. The resulting measurement model demonstrated improved fit to the data, *χ*^2^_(308)_ = 972.930, *χ*^2^/df = 3.16, RMSEA = 0.054, SRMR = 0.041, CFI = 0.956, and TLI = 0.949. As a robustness check, the structural model was re-estimated without this residual covariance. Although the inclusion of the covariance improved overall model fit, the substantive structural estimates and their pattern of statistical significance remained essentially unchanged.

Convergent and discriminant validity were subsequently examined. As shown in Table 4, AVE values ranged from 0.60 to 0.79, exceeding the recommended threshold of 0.50, while CR values ranged from 0.86 to 0.96, exceeding the recommended threshold of 0.70 (Hair et al., 2019). HTMT values ranged from 0.159 to 0.642, all below the recommended threshold of 0.90 (Henseler et al., 2015), supporting discriminant validity among the study constructs.

Taken together, these findings supported the convergent and discriminant validity and overall adequacy of the measurement model, justifying progression to the structural model analysis.

#### 3.2.3. Structural Model Results

**Figure 2:**
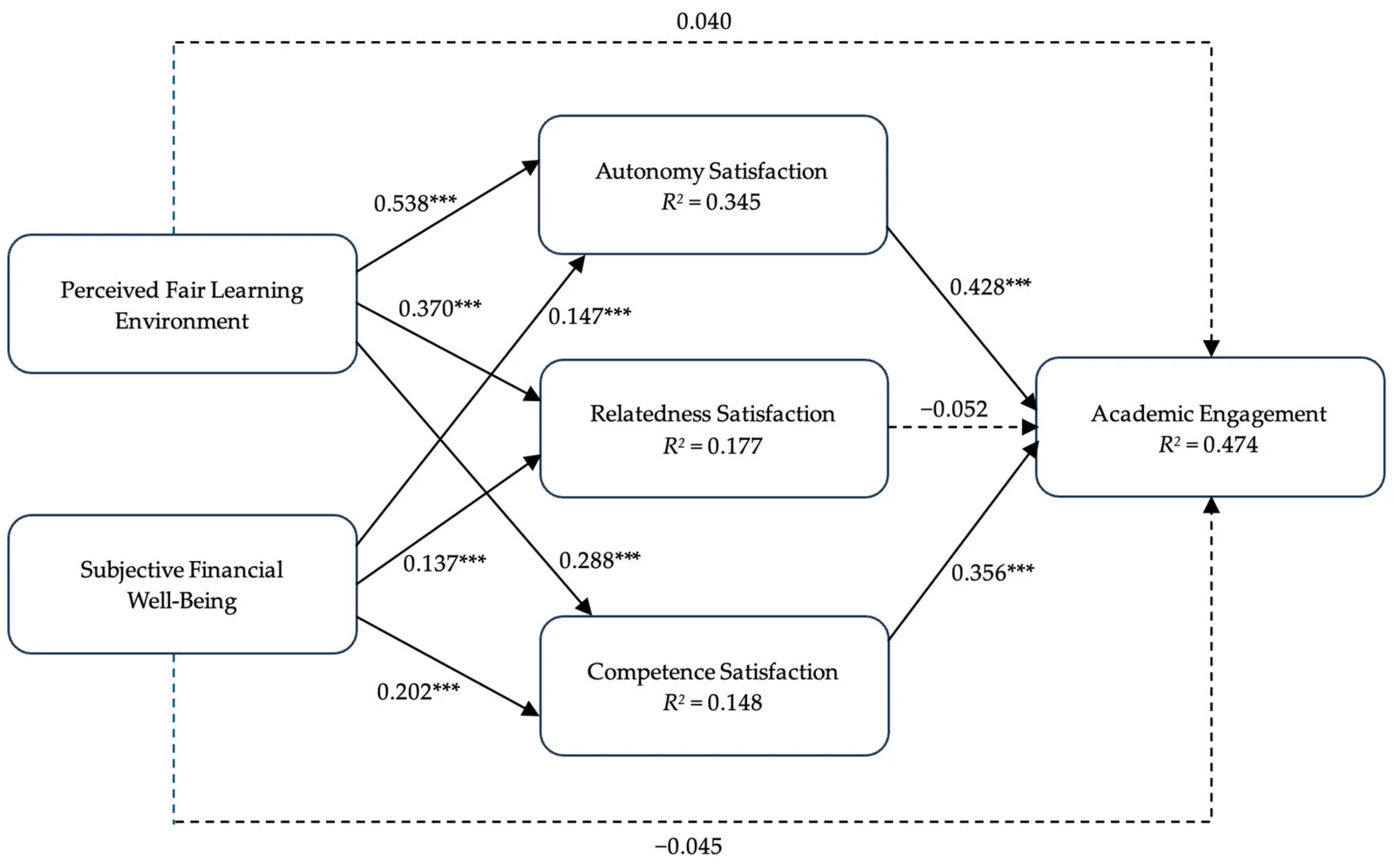
Structural model results. *Note.* Standardized path coefficients are shown. Solid lines indicate statistically significant paths, whereas dashed lines indicate non-significant paths. *R*^2^ values indicate the proportion of variance explained in the endogenous constructs. The disturbances of autonomy, relatedness, and competence satisfaction were allowed to covary. Measurement indicators are omitted for clarity. *** *p* < 0.001.

The hypothesized structural model was evaluated using structural equation modeling. Because the structural portion of the model was saturated, the structural model yielded the same global fit indices as the measurement model (*χ*^2^_(308)_ = 972.930, *χ*^2^/df = 3.16, RMSEA = 0.054, SRMR = 0.041, CFI = 0.956, and TLI = 0.949). The model explained 34.5% of the variance in autonomy satisfaction (*R*^2^ = 0.345), 17.7% in relatedness satisfaction (*R*^2^ = 0.177), 14.8% in competence satisfaction (*R*^2^ = 0.148), and 47.4% in academic engagement (*R*^2^ = 0.474). A graphical summary of the structural model results is provided in Figure 2.

Table 5 presents the structural path estimates. Perceived fair learning environment positively predicted autonomy satisfaction (*β* = 0.538, 95% BC CI [0.457, 0.610]), relatedness satisfaction (*β* = 0.370, 95% BC CI [0.290, 0.451]), and competence satisfaction (*β* = 0.288, 95% BC CI [0.204, 0.368]), supporting H1. Similarly, subjective financial well-being positively predicted autonomy satisfaction (*β* = 0.147, 95% BC CI [0.073, 0.220]), relatedness satisfaction (*β* = 0.137, 95% BC CI [0.062, 0.212]), and competence satisfaction (*β* = 0.202, 95% BC CI [0.120, 0.283]), supporting H2.

Autonomy satisfaction (*β* = 0.428, 95% BC CI [0.326, 0.527]) and competence satisfaction (*β* = 0.356, 95% BC CI [0.266, 0.444]) positively predicted academic engagement. In contrast, relatedness satisfaction was not significantly associated with academic engagement when the other two need dimensions were simultaneously accounted for (*β* = −0.052, 95% BC CI [−0.135, 0.026]). Accordingly, H3 was partially supported. After accounting for the three need satisfaction dimensions, neither the direct effect of perceived fair learning environment on academic engagement (*β* = 0.040, 95% BC CI [−0.055, 0.131]) nor that of subjective financial well-being (*β* = −0.045, 95% BC CI [−0.111, 0.024]) was significant. These direct paths were estimated to assess residual direct associations, although no directional hypotheses were specified for them.

Table 6 presents the specific indirect effects estimated using bias-corrected bootstrap confidence intervals based on 5000 resamples. Perceived fair learning environment had significant indirect effects on academic engagement through autonomy satisfaction (*B* = 0.183, 95% BC CI [0.132, 0.248]) and competence satisfaction (*B* = 0.082, 95% BC CI [0.053, 0.118]), whereas the indirect effect through relatedness satisfaction was not significant (*B* = −0.015, 95% BC CI [−0.041, 0.007]). Accordingly, **H4** was partially supported. A similar pattern emerged for subjective financial well-being: the indirect effects through autonomy satisfaction (*B* = 0.033, 95% BC CI [0.017, 0.055]) and competence satisfaction (*B* = 0.038, 95% BC CI [0.022, 0.059]) were significant, whereas the indirect effect through relatedness satisfaction was not (*B* = −0.004, 95% BC CI [−0.012, 0.001]). Thus, **H5** was partially supported.

The total standardized indirect effect of perceived fair learning environment on academic engagement through the three need satisfaction dimensions was significant (*β* = 0.313, 95% BC CI [0.240, 0.390]). The corresponding total indirect effect of subjective financial well-being was also significant (*β* = 0.128, 95% BC CI [0.076, 0.180]). Thus, both resources were indirectly associated with academic engagement, but these associations were carried specifically through autonomy and competence satisfaction rather than relatedness satisfaction. The estimated total indirect effect was larger for perceived fair learning environment than for subjective financial well-being.

As a sensitivity analysis, the same structural model was re-estimated using the full second sample (*N* = 755), retaining the 14 observations identified as outliers. The model using the full second sample also demonstrated acceptable fit, *χ*^2^_(308)_ = 1024.524, *χ*^2^/df = 3.33, RMSEA = 0.056, SRMR = 0.044, CFI = 0.955, and TLI = 0.949. The standardized path estimates and explained variance were highly similar to those obtained in the screened sample, and the significance pattern of the direct and indirect effects remained unchanged.

## 4. Discussion

This study examined whether autonomy, relatedness, and competence satisfaction mediate the relationships of perceived fair learning environment and subjective financial well-being with academic engagement. The findings provided full support for H1 and H2 and partial support for H3–H5. Both resources positively predicted all three dimensions of need satisfaction; however, only autonomy and competence satisfaction uniquely predicted academic engagement, whereas relatedness satisfaction did not. Consistent with this pattern, both perceived fair learning environment and subjective financial well-being had significant indirect effects on academic engagement through autonomy and competence satisfaction, but not through relatedness satisfaction. The model explained 34.5% of the variance in autonomy satisfaction, 17.7% in relatedness satisfaction, 14.8% in competence satisfaction, and 47.4% in academic engagement. Overall, perceived fair learning environment showed a stronger indirect association with academic engagement than subjective financial well-being, although the indirect associations of both resources were carried by the same two need dimensions, autonomy and competence satisfaction.

The strong predictive role of a fair learning environment in need satisfaction highlights the role that institutional conditions can play in supporting the fulfillment of students’ basic psychological needs. When all three need satisfaction dimensions were included in the model, the fair learning environment no longer showed a direct association with academic engagement; instead, significant indirect associations emerged through autonomy and competence satisfaction. On this evidence, the association between institutional fairness and engagement is statistically accounted for by students’ appraisals of the conditions they encounter. The belongingness literature helps clarify one aspect of this finding. What a fair environment conveys to a student is a set of cues about how far they are seen as a valued and accepted member of the academic community (Strayhorn, 2019; Tinto, 2017). A related pattern appears in the study by Główczewski and Burdziej (2023). Although the mediators differ, both studies indicate that the relation between fairness and academic engagement may not be a matter of direct connection alone, and that it may take shape through psychological processes arising in the course of a student’s academic life. The finding also extends Xin’s (2022) result that perceived social support predicts engagement through need satisfaction to a condition situated at the institutional level.

The associations of a fair learning environment with the three needs were unevenly distributed, being stronger for autonomy satisfaction and more moderate for competence satisfaction, whereas the coefficients through which subjective financial well-being predicted the three need satisfaction dimensions were distributed fairly evenly. These findings suggest that the conduct of the institution may be more closely linked to a student’s sense of directing their own actions, while also indicating that material conditions may be associated with the different dimensions of need satisfaction to a relatively similar extent. A further important finding is that financial well-being was not directly associated with academic engagement. This finding supports the expectation that students’ material circumstances may also be related to their motivational processes. Subjective financial well-being was associated with academic engagement indirectly through students’ experiences of competence and autonomy. These results are consistent with evidence that the need satisfaction process operates equivalently across socioeconomic status groups (Rodríguez-Meirinhos et al., 2020) and with studies showing that the relation between material position and academic outcomes is accounted for by psychological states (Ye et al., 2021). The relatively more modest indirect associations of subjective financial well-being do not diminish the importance of the shared motivational pathway. Indeed, Di Domenico and Fournier (2014) reported a similar pattern of effect sizes for material indicators in the general population.

Of the three need satisfaction dimensions, only relatedness satisfaction did not make a unique contribution to academic engagement. This finding suggests that relatedness satisfaction may not explain additional variance in academic engagement once students’ autonomy and competence satisfaction are taken into account. This finding is consistent with evidence suggesting that the needs do not follow a fixed hierarchy and that which need becomes more salient depends on the outcome under consideration (Bureau et al., 2022; Chen et al., 2015). The outcome measured here is the energy, dedication and absorption a student directs toward their own work (Schaufeli et al., 2002), not their participation in the academic community. The literature indeed shows that relatedness becomes more prominent when the outcome under consideration changes (Lecy, 2021; Tinto, 2017).

Liu et al. (2024) found that among university students, only relatedness satisfaction mediated the relation between teacher confirmation and academic engagement, whereas autonomy and competence satisfaction did not. This result is the reverse of the pattern obtained here. Taken together, the two findings suggest that which need becomes more prominent may vary with the context studied and with the character of the outcome. This difference can be made sense of by starting from the distinction Self-Determination Theory draws between the needs. Ryan and Deci (2017) note that autonomy and competence satisfaction are particularly closely linked to individuals’ sense of voluntarily directing their own actions and feeling effective, whereas relatedness plays an important part in the internalization of the values and behaviors of the social environment. The adoption of socially transmitted values is also shaped by the quality of the bonds formed with the people and groups who carry them. The academic engagement measured here emphasizes the energy, dedication, and absorption students direct toward their own academic work. The engagement Liu et al. (2024) examined in foreign language classrooms, by contrast, is tied more closely to classroom interaction and to the relationship formed with the teacher. On this account, what determines which need will carry the relation with academic engagement may depend on the particular form and context of engagement being examined. This possibility can be tested directly in studies that compare different forms of engagement within a single model.

### 4.1. Theoretical Implications

The pattern of findings offers several important theoretical implications. The first concerns the internal structure of the common process. The commonality was found only in autonomy and competence satisfaction. The three needs therefore do not make equivalent unique contributions to academic engagement. This distinction is also consistent with the distinction drawn within Self-Determination Theory itself. Ryan and Deci (2017) position autonomy and competence as proximal influences on intrinsic motivation, whereas relatedness is described as playing an important role in internalization. Accordingly, which needs carry the association between resources and engagement may vary depending on the nature of the outcome and should be determined empirically. Because a composite need satisfaction score would obscure this distinction, modeling the three needs separately is not only a methodological choice but also an important analytical condition for testing the proposed common-pathway expectation.

The second concerns the scope of the common motivational process. Basic Psychological Needs Theory already proposes that environmental conditions are related to individuals’ functioning through need satisfaction and links the status of a need as basic to the extent to which this account holds across a broad range of conditions (Vansteenkiste et al., 2020). The contribution of the present study lies in extending the range of conditions under which this claim is examined. One of the resources examined here is students’ perception of an institutional condition, the other a condition of life outside the study environment, and resources of this second kind have so far gone unexamined in the SD-R literature (Stoffels & Pienaar, 2026). Although the two resources differed in the strength of their associations with the three needs, both were indirectly associated with academic engagement through the same two needs, suggesting that this commonality does not stem from similarities between the resources themselves. Seen in this way, the distinction SD-R draws between contextual and personal resources sorts the inputs, and does not by itself determine the content of the motivational process through which those inputs operate.

The findings also contribute separately to each of the two bodies of literature on which the study draws. In the educational justice literature, the association between a fair learning environment and academic engagement can be given a theoretically specified psychological account rather than being treated simply as a correlation. This contribution gains further significance in light of a related observation concerning instruments used to assess the learning environment in higher education. In a scoping review covering sixty-eight studies, forty-five of them stated no explicit theoretical framework (Stoffels & Pienaar, 2026). Placing Lizzio et al.’s (2007) two-factor instrument within Basic Psychological Needs Theory provides a theoretical explanation for why students’ judgments of the learning environment may matter for engagement. Subjective financial well-being, for its part, has been studied largely in relation to its predictors, and its documented outcomes remain confined to subjective well-being, depressed mood, academic achievement and physical health (Sorgente & Lanz, 2017). It enters the academic engagement literature here as an indicator of how differences in material position may find a counterpart in students’ motivational lives. The model suggests that students’ subjective appraisals of their financial circumstances may be linked to academic engagement through their experiences of need satisfaction.

The findings also speak to a theoretical question that remains open in the literature. It remains contested whether justice should be understood as a basic psychological need in its own right or as a contextual condition that shapes the fulfillment of basic psychological needs. Molinari and Mameli (2018), working with secondary school students, treated fairness as a distinct need dimension and reported that the need for justice accounted for incremental variance in emotional and behavioral engagement over and above the other needs, while showing no such contribution to cognitive engagement. For agentic engagement the contribution was negative. On this basis they argued that fairness should be treated as an additional basic psychological need in school settings. Autonomy, however, did not emerge as a coherent factor in that sample and was replaced by a dimension of low tension. Thus, the set of needs against which justice was compared did not correspond to the canonical three. The findings of the present study are instead more consistent with a position that treats justice as a contextual condition supporting the fulfillment of existing basic psychological needs rather than extending the set of needs to include justice as an independent need. When modeled as an antecedent, the fair learning environment positively predicted all three dimensions of need satisfaction and was the stronger of the two resource predictors. Nevertheless, because the two theoretical accounts were not directly compared in the present study, this finding does not constitute evidence against treating justice as an additional basic psychological need. What it does suggest is that explaining the motivational significance of justice may not require departing from the parsimony of the three-need structure.

### 4.2. Practical Implications

The implications below differ in how closely they follow from the data. Some restate associations observed in the model, whereas others are institutional applications that these associations make plausible but do not themselves test. Of the two resources examined in this study, a fair learning environment stands out as one that institutions can shape directly, and its indirect relation with academic engagement is considerably larger than that of subjective financial well-being. Since what was measured here is not institutional arrangements themselves but students’ perceptions of those arrangements, it should not be overlooked that in practice what matters is not only whether an arrangement exists but whether it reaches the student. Setting out assessment criteria, keeping channels for appeal and correction in working order, and giving reasons for decisions should therefore be treated as a matter of communication. The findings also give some direction as to which needs might be targeted. Given that autonomy and competence were the need dimensions that carried the indirect associations of the two resources with academic engagement, practices that give students a voice in decisions that affect them and provide clear and predictable procedures may be particularly relevant to supporting these needs. Because the two subdimensions of a fair learning environment were not modeled as separate latent variables, this suggestion calls for direct testing.

Some caution is warranted, by contrast, in assuming that belonging-focused interventions will necessarily translate into task-directed engagement. Relatedness satisfaction was positively correlated with academic engagement at the bivariate level in this study, but it accounted for no additional variance once autonomy and competence satisfaction were included in the model. The finding therefore concerns the absence of a unique association within this set of predictors and should not be read as evidence that relatedness is unimportant for students’ academic lives. Fernández et al. (2026), in a meta-analysis of five pre-registered experiments with 1485 students in STEM disciplines, found that students in the higher belonging condition were more likely to see advancement within academic hierarchies as attainable for themselves. Although the effect was small, as the authors also discuss, such perceptions of permeability may create conditions in which structural barriers are overlooked by emphasizing individual advancement. Belonging is a worthwhile aim in its own right where outcomes such as attachment to the institution and persistence are concerned. However, when it comes to task-directed engagement, the present findings point more directly to practices that support autonomy and competence.

The association between students’ financial circumstances and academic engagement through need satisfaction suggests that financial support may also matter for motivational processes. Scholarships and hardship funds may therefore serve a purpose beyond that of a means of access. Because it was students’ appraisals of their own financial position, not their material position in itself, that were associated with need satisfaction here (Di Domenico & Fournier, 2014), what an institution does about the ordinary costs of participation comes to matter alongside the resources it transfers. Heckman et al. (2014) found that the strongest predictor of financial stress among university students was lacking the means to take part in the same activities as their peers. Field trips, laboratory materials, printing, software, textbooks, and the small costs attached to academic life can each create a visible divide between students who can afford them without having to think about the expense and those who cannot. Incorporating these costs into the ordinary provision of academic activities may reduce the extent to which financial differences are reflected in students’ everyday academic experiences.

How support reaches students is also part of this function. Assistance administered through a separate unit, entailing a documentary burden and resting on discretionary decisions, may require students to present their circumstances as a deficiency. Given the relation of engagement to autonomy and competence satisfaction, procedures that leave students having to demonstrate their own inadequacy in order to obtain financial support may bear adversely on the very needs found to be related to engagement. Disbursement that is automatic or opt-out, eligibility criteria stated in advance, and decisions open to appeal may therefore help provide financial support in a form closer to an entitlement, one that does not require students to prove their own deficiency.

It is at this point that the two resources meet in practice. A university cannot change a student’s family income, but it can influence whether financial hardship is compounded by non-transparent procedures and inequitable practices, and this is an area in which institutions can intervene directly. Whether institutional arrangements and resources actually reach students’ experiences is a separate question that institutions can ask themselves. The recommendation to regularly assess study demands and resources (Bakker & Mostert, 2024) offers one way of addressing this issue, while need satisfaction may provide an indicator of whether these resources are reflected in students’ experiences.

None of the arrangements discussed here was examined in the present study. What the findings establish is a set of associations among fairness perceptions, financial well-being, need satisfaction and engagement. Whether changing these arrangements alters need satisfaction or engagement is a question for intervention and longitudinal research.

### 4.3. Limitations

These findings should be interpreted in light of several limitations. The first limitation concerns the research design and the direction of causality. The cross-sectional design does not allow the causal or temporal direction of the associations between the variables to be determined. The Study Demands–Resources approach, moreover, holds that the relation between resources and engagement may not be one-directional. Demands and resources interact and accumulate over time, and students who start with more resources tend to acquire more of them as time goes on (Salmela-Aro et al., 2022). Bakker and Mostert (2024) further propose that high engagement can itself set off a gain spiral of proactive study behaviors and resources, and they note that many of the paths in the model are reciprocal. The mediation structure proposed here should therefore be taken as a model that is theoretically grounded and consistent with the data, while the direction of causality and any reciprocal or spiraling dynamics should be tested with longitudinal, cross-lagged and experimental designs.

The second concerns measurement. Although the single-factor test did not indicate a serious common method bias concern, the reliance on self-report measures for all variables leaves open the possibility of common method variance. A fair learning environment was modeled as a single latent variable with two subdimensions. Therefore, the study did not test whether respectful partnership and systemic fairness have different associations with the three need satisfaction dimensions. The theoretical expectation is that the two dimensions feed different needs, and this was taken to be a question that further research could examine. In addition, subjective financial well-being was measured only through its general component, and the other components of the construct were not included in the model. The basic psychological needs, moreover, were measured only in terms of satisfaction, and need frustration was not brought into the model. Since Self-Determination Theory treats satisfaction and frustration as separate functional pathways that cannot be reduced to one another (Rodríguez-Meirinhos et al., 2020; Vansteenkiste et al., 2020), the findings are confined to the motivational process involving need satisfaction. Academic engagement, for its part, was approached in terms of task-directed energy, dedication and absorption. The finding concerning relatedness satisfaction may therefore be specific to engagement understood in this way.

## 5. Conclusions

Academic engagement, on the evidence assembled here, should not be seen as a property students simply bring to their studies. The conduct of an institution and the circumstances of students’ lives are related to academic engagement insofar as they are reflected in students’ experiences of autonomy and competence. Two resources of markedly different origin were related to academic engagement through the same two needs. The contribution of a fair learning environment was appreciably stronger, while that of subjective financial well-being was more modest. Both, however, were related to engagement only indirectly. Students do not enter the academic community from equivalent positions, and the model indicates one point at which that difference is reflected in students’ motivational lives. Universities do not determine the incomes their students arrive with, but much of what follows from those incomes once a student is inside the institution is a matter of institutional design, from what ordinary participation costs to how support is delivered. Because it is the student’s own appraisal that was associated with engagement here, these are matters institutions can work on. Fair practice and attention to the material conditions of student life stand among the conditions that matter for motivation toward academic work.

## Figures and Tables

**Table 1:** Demographic characteristics of the two samples.

Variable	Category	Sample 1 (*n* = 278)	Sample 2 (*n* = 741)
Gender	Female	205	524
	Male	66	212
	Not disclosed	7	5
Grade	Preparatory school	32	41
	1st year	48	120
	2nd year	77	250
	3rd year	87	243
	4th year or above	34	87
Age	Age (Mean)	21.2	21.5

Note. Sample 1 demographics are reported for the full sample (*n* = 278); scale-specific analytic sample sizes varied slightly after data screening (see Section 2.2). Sample 2 represents the final analytic sample (*n* = 741).

**Table 2:** Psychometric evaluation of the study measures.

Indicator	Perceived Fair Learning Environment	Financial Well-Being	Basic Psychological Need Satisfaction	Academic Engagement
*n*	271	278	259	268
Skewness	0.148	0.221	−0.453	0.278
Kurtosis	−0.291	−0.795	0.336	−0.304
*χ*^2^/*df*	2.433	2.605	2.529	2.526
RMSEA	0.073	0.076	0.077	0.076
SRMR	0.043	0.024	0.054	0.041
CFI	0.944	0.980	0.952	0.970
TLI	0.933	0.973	0.938	0.952
*α*	0.927	0.957	0.874	0.883
ω	0.928	0.957	0.874	0.888

Note. *n* = sample size; *χ*^2^/*df* = chi-square divided by degrees of freedom; RMSEA = root mean square error of approximation; SRMR = standardized root mean square residual; CFI = comparative fit index; TLI = Tucker–Lewis index; α = Cronbach’s alpha coefficient; ω = McDonald’s omega coefficient.

**Table 3:** Descriptive statistics, reliability estimates, correlations, and diagnostic indices among the study variables.

Variable	*M*	*SD*	*α*	*ω*	1	2	3	4	5	6
1. Perceived Fair Learning Environment	3.22	0.81	0.93	0.93	-					
2. Subjective Financial Well-Being	2.70	1.11	0.96	0.96	0.20 **	-				
3. Autonomy Satisfaction	3.37	0.83	0.85	0.85	0.54 **	0.24 **	-			
4. Relatedness Satisfaction	3.71	0.79	0.88	0.88	0.35 **	0.20 **	0.37 **	-		
5. Competence Satisfaction	3.67	0.76	0.87	0.87	0.29 **	0.24 **	0.51 **	0.30 **	-	
6. Academic Engagement	2.49	0.78	0.90	0.90	0.32 **	0.15 **	0.56 **	0.22 **	0.50 **	-
Skewness					−0.101	0.188	−0.449	−0.652	−0.576	0.425
Kurtosis					−0.338	−0.913	0.198	0.717	0.730	−0.135
TI					0.68	0.91	0.56	0.80	0.71	-
VIF					1.46	1.10	1.79	1.24	1.40	-

Note. *M* = mean; *SD* = standard deviation; *α* = Cronbach’s alpha coefficient; *ω* = McDonald’s omega coefficient; TI = tolerance index; VIF = variance inflation factor. ** *p* < 0.01.

**Table 4:** Convergent and Discriminant Validity of the Measurement Model.

Construct	AVE	CR	1	2	3	4	5	6
1. Perceived Fair Learning Environment	0.79	0.88	-					
2. Subjective Financial Well-Being	0.69	0.96	0.216	-				
3. Autonomy Satisfaction	0.60	0.86	0.627	0.264	-			
4. Relatedness Satisfaction	0.64	0.88	0.394	0.213	0.428	-		
5. Competence Satisfaction	0.62	0.87	0.335	0.250	0.595	0.343	-	
6. Academic Engagement	0.71	0.88	0.359	0.159	0.642	0.241	0.560	-

Note. AVE = average variance extracted; CR = composite reliability. Values below the diagonal are heterotrait–monotrait ratios of correlations (HTMT). Recommended thresholds are AVE ≥ 0.50, CR ≥ 0.70, and HTMT < 0.90.

**Table 5:** Structural path estimates.

H	Path	*β*	95% BC CI	Result
H1	Fair learning environment → Autonomy satisfaction	0.538	[0.457, 0.610]	Significant
	Fair learning environment → Relatedness satisfaction	0.370	[0.290, 0.451]	Significant
	Fair learning environment → Competence satisfaction	0.288	[0.204, 0.368]	Significant
H2	Subjective financial well-being → Autonomy satisfaction	0.147	[0.073, 0.220]	Significant
	Subjective financial well-being → Relatedness satisfaction	0.137	[0.062, 0.212]	Significant
	Subjective financial well-being → Competence satisfaction	0.202	[0.120, 0.283]	Significant
H3	Autonomy satisfaction → Academic engagement	0.428	[0.326, 0.527]	Significant
	Relatedness satisfaction → Academic engagement	−0.052	[−0.135, 0.026]	Not significant
	Competence satisfaction → Academic engagement	0.356	[0.266, 0.444]	Significant
-	Fair learning environment → Academic engagement	0.040	[−0.055, 0.131]	Not significant
-	Subjective financial well-being → Academic engagement	−0.045	[−0.111, 0.024]	Not significant

Note. *β* = standardized path coefficient; BC CI = bias-corrected confidence interval based on 5000 bootstrap resamples.

**Table 6:** Specific Indirect Effects.

H	Path	*B*	95% BC CI	Result
H4	Fair learning environment → Autonomy → Academic engagement	0.183	[0.132, 0.248]	Significant
	Fair learning environment → Relatedness → Academic engagement	−0.015	[−0.041, 0.007]	Not significant
	Fair learning environment → Competence → Academic engagement	0.082	[0.053, 0.118]	Significant
H5	Subjective financial well-being → Autonomy → Academic engagement	0.033	[0.017, 0.055]	Significant
	Subjective financial well-being → Relatedness → Academic engagement	−0.004	[−0.012, 0.001]	Not significant
	Subjective financial well-being → Competence → Academic engagement	0.038	[0.022, 0.059]	Significant

Note. *B* = unstandardized specific indirect effect; BC CI = bias-corrected confidence interval based on 5000 bootstrap resamples.

## Data Availability

The anonymized analytic datasets supporting the findings of this study is publicly available in Zenodo at https://doi.org/10.5281/zenodo.22063031.

## Supplementary Materials

The following supporting information can be downloaded at: https://www.mdpi.com/article/10.3390/bs16101806/s1, Basic Psychological Need Satisfaction Scale: Academic Adaptation of the BPNSFS Need Satisfaction Items, including information on the adaptation procedure, scale structure, response format, and the adapted Turkish items with their English translations.

## Abbreviations

The following abbreviations are used in this manuscript:

BC CI	Bias-Corrected Confidence Interval
BPNSFS	Basic Psychological Need Satisfaction and Frustration Scale
BPNT	Basic Psychological Needs Theory
CFA	Confirmatory Factor Analysis
CFI	Comparative Fit Index
CR	Composite Reliability
HTMT	Heterotrait–Monotrait Ratio of Correlations
JD-R	Job Demands–Resources
RMSEA	Root Mean Square Error of Approximation
SD-R	Study Demands–Resources
SDT	Self-Determination Theory
SEM	Structural Equation Modeling
SRMR	Standardized Root Mean Square Residual
TI	Tolerance Index
TLI	Tucker–Lewis Index
UWES-SF	Utrecht Work Engagement Scale–Student Form
VIF	Variance Inflation Factor
