# Supplementary material for: Fair Learning Environment and Financial Well-Being as Resources for Students’ Academic Engagement: Basic Psychological Need Satisfaction as a Common Motivational Pathway
Source: Behav Sci (Basel). 2026 Oct 2;16(10):1806. doi: 10.3390/bs16101806 (PMC13701982; doi:10.3390/bs16101806)
Supplement: Supplementary file 1 [file behavsci-16-01806-s001.zip › behavsci-4587941-supplementary.pdf]

Basic Psychological Need Satisfaction Scale: Academic Adaptation of the BPNSFS Need Satisfaction Items

The scale was adapted from the 12 need satisfaction items of the Basic Psychological Need Satisfaction and Frustration Scale (BPNSFS; Chen et al., 2015) and contextualized to the academic experiences of university students. It comprises three four-item subscales: *autonomy satisfaction* (Items 1–4), *relatedness satisfaction* (Items 5–8), and *competence satisfaction* (Items 9–12). Items were administered to university students in Turkish. An English version was prepared for international readers through forward translation, back-translation, and expert committee review procedures to ensure conceptual equivalence. The English version is provided for reference purposes and has not been independently psychometrically validated. Need frustration items were not included. Responses are rated on a five-point Likert-type scale (1 = strongly disagree, 2 = disagree, 3 = neither agree nor disagree, 4 = agree, and 5 = strongly agree).

Items	Turkish (Administered)	English (For Reference)
**Autonomy Satisfaction**		
**Item 1**	Üniversite eğitimimde üstlendiğim çalışmaları özgürce seçebildiğimi hissederim.	In my university education, I feel a sense of choice and freedom in the work I undertake.
**Item 2**	Eğitimimle ilgili kararlarımın gerçekten ne istediğimi doğru yansıttığını hissederim.	I feel that my decisions about my education reflect what I really want.
**Item 3**	Akademik tercihlerimin kim olduğumu doğru yansıttığını hissederim.	I feel that my academic choices express who I really am.
**Item 4**	Üniversite eğitimimde gerçekten ilgimi çeken şeyler üzerinde çalıştığımı hissederim.	In my university education, I feel I have been doing what really interests me.
**Relatedness Satisfaction**		
**Item 5**	Üniversitede önemsediğim insanların da beni önemsediğini hissederim.	At university, I feel that the people I care about also care about me.
**Item 6**	Üniversitede önemsediğim ve beni önemseyen insanlarla aramda bağ kurulmuş hissederim.	At university, I feel connected with people who care for me, and for whom I care.
**Item 7**	Üniversitede benim için önemli olan insanlara kendimi yakın ve bağlı hissederim.	At university, I feel close and connected with other people who are important to me.
**Item 8**	Üniversitede birlikte zaman geçirdiğim kişilere karşı sıcaklık ve yakınlık hissederim.	At university, I experience a warm feeling with the people I spend time with.
**Competence Satisfaction**		
**Item 9**	Akademik görevleri layıkıyla yapabileceğim konusunda kendime güvenirim.	I feel confident that I can do academic tasks well.
**Item 10**	Derslerimde kendimi yeterli hissederim.	I feel capable in my courses.
**Item 11**	Akademik hedeflerime ulaşmak için yeterli olduğumu hissederim.	I feel competent to achieve my academic goals.
**Item 12**	Zor akademik görevleri başarıyla tamamlayabileceğimi hissederim.	I feel I can successfully complete difficult academic tasks.

Use note. This adaptation is intended for academic, non-commercial use. The terms of use of the original BPNSFS apply.

Reference

Chen, B., Vansteenkiste, M., Beyers, W., Boone, L., Deci, E. L., Van der Kaap-Deeder, J., Duriez, B., Lens, W., Matos, L., Mouratidis, A., Ryan, R. M., Sheldon, K. M., Soenens, B., Van Petegem, S., & Verstuyf, J. (2015). Basic psychological need satisfaction, need frustration, and need strength across four cultures. *Motivation and Emotion, 39*, 216–236. https://doi.org/10.1007/s11031-014-9450-1

Use note. This adaptation is intended for academic, non-commercial use. The terms of use of the original BPNSFS apply.
